# Subviral Dense Bodies of Human Cytomegalovirus Enhance Interferon-Beta Responses in Infected Cells and Impair Progeny Production

**DOI:** 10.3390/v15061333

**Published:** 2023-06-07

**Authors:** Inessa Penner, Nicole Büscher, Steffi Krauter, Bodo Plachter

**Affiliations:** Institute for Virology, University Medical Center of the Johannes Gutenberg-University Mainz, D-55131 Mainz, Germany; inpenner@uni-mainz.de (I.P.); bueschni@uni-mainz.de (N.B.); krauter@uni-mainz.de (S.K.)

**Keywords:** human cytomegalovirus, subviral particles, dense bodies, apoptosis, interferon-β, IRGs

## Abstract

(1) Background: Infection with human cytomegalovirus (HCMV) leads to the production and release of subviral particles, termed Dense Bodies (DB). They are enclosed by a membrane resembling the viral envelope. This membrane mediates the entrance of DBs into cells in a way that is comparable to virus infection. HCMV attachment and entry trigger the induction of interferon synthesis and secretion, and the subsequent expression of interferon-regulated genes (IRGs) that might inhibit replication of the virus. Recently, we demonstrated that DBs induce a robust interferon response in the absence of infection. Little is known thus far, including how DBs influence HCMV infection and virus–host interaction. (2) Methods: Purified DBs were used to study the impact on virus replication and on the innate defense mechanisms of the cell. (3) Results: The incubation of cells with DBs at the time of infection had little effect on viral genome replication. Preincubation of DBs, however, led to a marked reduction in viral release from infected cells. These cells showed an enhancement of the cytopathic effect, associated with a moderate increase in early apoptosis. Despite virus-induced mechanisms to limit the interferon response, the induction of interferon-regulated genes (IRGs) was upregulated by DB treatment. (4) Conclusions: DBs sensitize cells against viral infection, comparable to the effects of interferons. The activities of these particles need to be considered when studying viral–host interaction.

## 1. Introduction

The human cytomegalovirus (HCMV) is a pathogen of high medical relevance. Congenital HCMV infection frequently leads to severe manifestations and sequelae [1,2,3]. Viral reactivation in patients receiving solid organ or hematopoietic stem cell transplants may cause life-threatening conditions [4,5]. Thus, the development of both therapeutic as well as prophylactic strategies against HCMV disease is of major research interest. For this, it is pivotal to understand the interaction of the virus with its host cell. A significant body of literature has been published on this topic. One aspect that has not been well considered is the question about the impact of non-infectious viral particles on viral infection. It is well established that permissively infected culture cells release not only infectious progeny, but also at least two forms of enveloped viral particles that are not infectious. The NIEPs, or non-infectious enveloped particles, display a similar structure to virions, but lack viral DNA. They harbor one additional protein termed scaffold protein, which is removed from infectious virions in the course of capsid assembly [6,7]. The Dense Bodies (DBs) are electron-dense structures found in the cytoplasm of infected cells that lack viral genomes or capsids [8,9,10]. These DBs are released in large amounts from culture cells and can be separated by either gradient ultracentrifugation or by ultrafiltration [6,11,12]. Because of their immunogenic properties, DBs have been denoted as a promising vaccine candidate [11,13,14,15,16,17,18]. Interestingly, DBs were also found in vivo, demonstrating that the synthesis of these particles was not a cell culture artefact [19].

Cell culture supernatants that are frequently used for HCMV experiments contain large numbers of DBs [20]. In our previous study, we demonstrated that the treatment of fibroblasts or endothelial cells with DBs induced a set of interferon responsive genes (IRGs). This was strictly dependent on interferon-β (IFN-β) secretion and on interferon regulatory factor 3 (IRF3) expression [21]. As HCMV replication is sensitive to the IFN-β response, this raises the question about a possible impact of these particles on the results of such experiments and the role of DB in HCMV infection in general. We addressed this issue here by adding purified DBs to HCMV-infected cells. Surprisingly, viral progeny production was reduced after the DBs preincubation of cell cultures. This correlated with moderately increased early apoptosis and an enhanced induction of interferon-regulated genes (IRGs).

## 2. Materials and Methods

### 2.1. Cells and Viruses

Primary human foreskin fibroblasts (HFF) were propagated in minimal essential medium (MEM; PAA, Coelbe, Germany), supplemented with 5% or, for the apoptosis experiments, in 10% fetal calf serum (FCS; Biochrom, Berlin, Germany), 2 mM L-glutamin, 50 mg/L gentamycin and 0.5 ng/mL basic fibroblast growth factor (bFGF; ThermoFisher Scientific, Darmstadt, Germany). All HCMV strains used in this analysis were derived from bacterial artificial chromosome (BAC) clones. For HCMV infections, supernatant stocks of the endotheliotropic strains Towne-UL130repΔGFP (denoted herein as TR-∆GFP; [15]) and TB40/E-BAC7 (denoted herein as TB40/E; [22]) were used. The KB14 strain, lacking the pp65 gene [23], was used as a laboratory strain that does not produce DBs. Virus supernatant stocks were obtained by collecting supernatants from infected HFF cultures at 7 days post-infection (d.p.i.). The super natants were harvested and precleared from cellular debris by centrifugation at 1475× *g* for 10 min and then stored at −80 °C until further use.

### 2.2. Purification of DB and UV Inactivation

DBs of HCMV were prepared as previously described [24]. Briefly, HFFs were infected with virus supernatant stock TR-ΔGFP in the presence of 50 nM of letermovir (LMV). LMV was added to the cell culture media at the time of infection and 3 days after initial infection. Supernatants from infected HFFs that showed a complete CPE were harvested and gross cellular debris was removed by centrifugation for 10 min at 1475× *g* in 50 mL tubes. Afterwards, viral particles were pelleted via ultracentrifugation and fractionated via glycerol–tartrate density gradient ultracentrifugation [25]. Subsequently, the DB-fraction was visualized by light scattering and collected from the tube with a syringe. DBs were concentrated by ultracentrifugation and stored at −80 °C until further use. For the determination of DB-protein concentrations, the Pierce™ BCA Protein Assay Kit (cat.no. 23225, ThermoFisher Scientific, Darmstadt, Germany) was used according to the manufacturer’s protocol.

Purified DBs were irradiated with ultra-violet (UV) light at a wavelength of 254 nm before they were applied to cells. Depending on the experiment, the appropriate amount of DBs was thawed and dispersed in PBS. For UV irradiation, a spot plate was used. DBs were resuspended in PBS in a total volume of 120 µL and transferred onto a spot plate. Following irradiation with UV light for two minutes, 100 µL of the UV-irradiated DB/PBS solution was mixed with culture medium and added to the cells.

### 2.3. Application of Virus Supernatants and DBs to Cells

One day before infection or DB application, 0.5 × 10^6^ HFF cells were seeded on 10 cm dishes. For infection, virus supernatant stocks were diluted in 3 mL 5% MEM medium and applied to the cells. After 2 h inoculation, cells were washed twice with PBS and 10 mL of fresh 5% medium was added. Cells were incubated at 37 °C and 5% CO_2_ for the appropriate time. For the penetration of DBs into HFFs, DBs were diluted in 120 μL PBS and subjected to UV irradiation. Depending on the experiment, 100 µL of UV-irradiated DBs was either added to virus inoculum, or cells were preincubated with DBs before infection. For the pretreatment of cells with DB, 100 µL of UV-irradiated DBs was diluted in 2900 μL of 5% MEM and applied to cells for 2 h. Afterwards, the cells were washed twice with PBS and then infected.

### 2.4. Quantitative Real-Time Polymerase Chain Reaction (TaqMan qPCR) and Quantification of Viral Progeny

Quantitative real-time polymerase chain reaction (qPCR) using the TaqMan^®^ technology and quantification of viral progeny by limiting the dilution and IE1 protein staining were performed as described before [26].

### 2.5. Antibodies and Reagents

The following primary antibodies were used: the antibody p63-27, provided by William Britt, was used for IE1-staining [27,28]. Anti—IFIT3 (1:3000, PA5-22230, ThermoFisher Scientific, Darmstadt, Germany), anti—ISG15 (F-9) (1:500, sc-166755, Santa Cruz Biotechnology Inc., Dallas, TX, USA), anti—MX1 (1:1000, PA5-22101, ThermoFisher Scientific, Darmstadt, Germany), anti—pp65 (65-33, provided by William Britt, University of Birmingham, Birmingham, AL, USA), anti—Tubulin-alpha (DM1A) (1:500, T6199, Sigma-Alrich, Saint Louis, MO) and anti—UL44 (BS510) (Biotest AG, Dreieich, Germany) were used. As secondary antibodies, donkey anti-Rabbit Alexa Fluor™ 680 (1:10,000, A10043, ThermoFisher Scientific, Darmstadt, Germany) and IRDye^®^ 800CW donkey anti-mouse antibodies (1:15,000, 926-32212, LI-COR Biotechnology, Bad Homburg, Germany) were used. The recombinant human IFN-β was purchased from PeproTech, Hamburg, Germany (100 U/mL, #300-02BC). The inhibitor of the Janus protein tyrosine kinases (JAKs), JAK Inhibitor I, was purchased from Merck Millipore, Darmstadt, Germany (20 µm/mL, #420099).

### 2.6. Sodium Dodecyl Sulfate Polyacrylamide Gel Electrophoresis (SDS-PAGE) and Immunoblot Analysis

For immunoblot analysis, whole-cell protein lysates from infected or DB-treated cells were suspended in 2 × Laemmli buffer and heated for 10 min at 99 °C. Equal amounts of cell lysate (2 × 10^5^ cells) were loaded on Bolt™ 10% Bis-Tris gels (NW00105BOX, ThermoFisher Scientific, Darmstadt, Germany). Following electrophoresis, the proteins were transferred to a polyvinylidene difluoride membrane (PVDF; Immobilon-PSQ, KgaA, ISEQ00010, Merck, Darmstadt, Germany) using a Mini Blot module (NW2000, ThermoFisher Scientific, Darmstadt, Germany) at 20 V for 1 h. Afterwards, the membranes were blocked in a 5% non-fat dry milk solution prepared in Tris-buffered saline with 0.2% Tween 20 detergent (TBST) for 1 h. The incubations with primary antibodies were performed overnight at 4 °C on a roller mixer. Membranes were washed three times for 10 min with 0.2% TBST before they were incubated with secondary antibodies in 0.2% TBST for 2 h and were protected from the light at RT. After another three washes with 0.2% TBST for 10 min, protein bands were detected by an Odyssey Infrared Imager CLx (LI-COR Biotechnology, Bad Homburg, Germany).

### 2.7. FACS Analysis

Flow cytometry analysis was used to monitor apoptotic, necrosis and healthy cells after infection and DB application using the Apoptosis/Necrosis Detection Kit (Ab176749, Abcam, Cambridge, UK). HFFs were seeded in 10 cm dishes at a density of 0.5 × 10^6^ one day before infection and DB application. Prior to infection, the cells of two dishes were primed with 20 µg of UV-irradiated DBs/dish of the HCMV strain TR-ΔGFP for two hours. Following incubation, the virus supernatant, normalized for an uptake of 50 viral genomes per cell, was added and incubated for another two hours. In parallel, two dishes were inoculated with 50 genomes/cells of virus supernatant, two dishes with 50 genomes/cell virus supernatants and 20 µg of UV-irradiated DBs and the cells of two dishes were exposed to 20 µg of UV-irradiated DBs only. The inocula were allowed to adsorb for two hours at 37 °C and 5% CO_2_. Afterwards, the volume per dish was adjusted to 10 mL with 10% MEM medium and the cells were incubated for a further four hours. After a total incubation period of six hours, the inoculum was removed, and the cells were washed twice with PBS. Finally, the cells were maintained in fresh MEM medium containing 10% FCS for six days. On the day of the flow cytometry assay, untreated and treated cells were collected on ice. Before the staining solution, comprising 200 µL of assay buffer, 2 µL of Apopxin Green Indicator (100×) and 1 µL of 7-AAD (200×) per sample were applied to the cells and a master mix was prepared to ensure the same amount of the dye was placed in each sample. Half of the cells were stained using the respective primary antibody, while the rest were mock stained with FACS buffer, which was included in the kit to detect secondary antibody background staining. Cells were resuspended in 204 µL staining solution and staining was performed for 40 min at room temperature protected from light. To increase the volume before flow cytometer analysis, 300 µL of assay buffer was added to each sample. In addition, 1.5 × 10^5^ cells for each condition were recorded for the analysis on an FACS Cytomics FC 500 (Becton Dickinson) flow cytometer. For the quantification of Apopxin Green Indicator binding, the FL1 channel (Ex/Em = 490/525 nm) was used. 7-AAD was measured by using the FL3 channel (Ex/Em = 546/647). The data were analyzed using the corresponding BD CXP analysis software (Beckman Coulter Inc., 2006).

### 2.8. Statistical Analysis

All statistical tests were performed using GraphPad Prism version 8.30 for Windows (GraphPad Software, San Diego, CA, USA).

## 3. Results

### 3.1. Incubation of HFFs with DBs Has Little Impact on Viral DNA Replication and Subtle Impact on Genome Release

**Figure 1:**
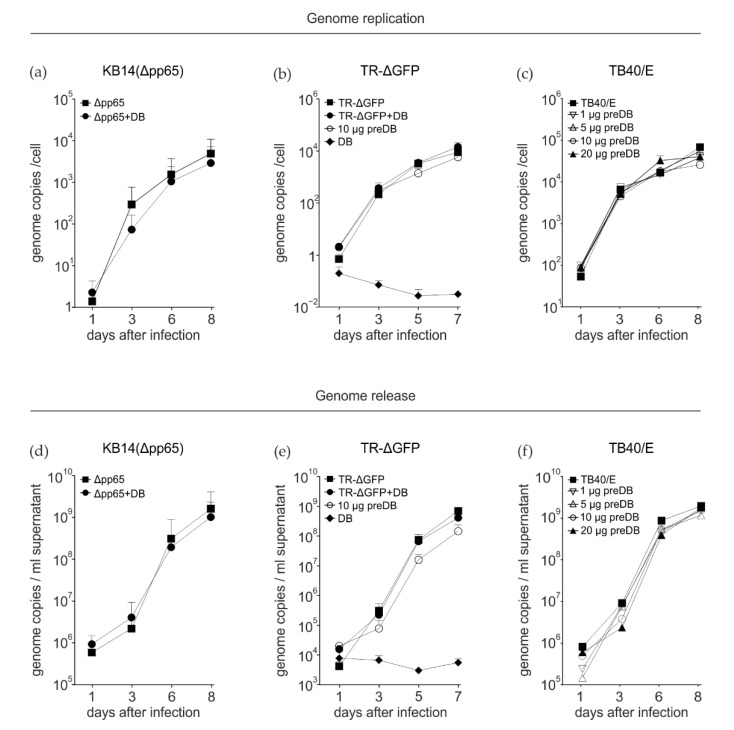
Impact of DBs on HCMV replication and genome release. Quantitative real time PCR (qRT-PCR) analysis of viral genome replication (**a**–**c**) and viral genome release (**d**–**f**) from HFF. (**a**) Cells were infected with 25 genomes per cell of strain KB14 (Δpp65, HCMV strain AD169 deletion mutant lacking the pp65 gene) or infected and concomitantly incubated with TR-ΔGFP-derived DBs (Δpp65+DB). (**b**) 10 μg of UV-inactivated DBs was added to HFF 2 h prior to infection (10 µg preDB) or simultaneously with TR-ΔGFP infection (TR-∆GFP+DB). As a control, HFFs were infected with TR-ΔGFP or only incubated with 10 μg of UV-inactivated DB alone (DB). (**c**) HFFs were primed with 1 to 20 µg of TR-ΔGFP DB for 2 h before infection with TB40/E (moi 0.5). Control cells were infected with TB40/E at a moi of 0.5. The cells were collected at the indicated time points. DNA from 10^5^ cells was isolated and the number of viral genomes was determined by qRT-PCR analysis. (**d**–**f**) HFFs were infected and DBs were applied as in a, b and c. Cell culture supernatants were collected at the indicated time points and cleared from cell debris by centrifugation. DNA from 200 μL of each supernatant was isolated and subjected to qRT-PCR analysis for genome determination. The data represent mean values ± SD of triplicate determinations from three (**a**,**b**,**d**,**e**) or two (**c**,**e**) independent experiments for each timepoint.

On the one hand, DB application to fibroblasts or endothelial cells elicits an antiviral state that is characterized by the expression of a subset of IRGs that are known for their antiviral properties [21,29,30] and on the other hand, DBs of HCMV contain proteins such as pp71 (pUL82) or pp65 (pUL83) that play an important role in regulating IE gene expression and viral replication [31,32,33,34,35]. It was thus interesting to examine if the addition of larger amounts of DBs concomitant with or before infection would either support or restrict HCMV replication. We consequently performed experiments to test this and initially chose to use an HCMV mutant that lacks the tegument protein pp65 (pUL83; KB14) [36]. Pp65 is essential for DB formation; therefore, the supernatant used for infection was DB-free. Cells were infected with 50 genomes/cell of KB14 by concomitantly adding 10 µg of UV-inactivated DB from the strain TR-ΔGFP. The latter strain was used for DB production, since DBs derived from TR-ΔGFP contain the pentameric complex of membrane proteins consisting of gH, gL, and pUL128-131, thus closely reflecting the wild-type situation [37]. HFFs were infected with HCMV KB14 and were concomitantly exposed to UV-inactivated DBs and analyzed by quantitative PCR (Figure 1a). No statistically significant differences were observed in the DB treatment regarding the genome replication of KB14, compared to the control. Since KB14 is lacking the PC, we next performed a similar experiment using TR-ΔGFP for infection. However, neither concomitant nor pre-DB application for 2 h before infection had an impact on viral genome replication (Figure 1b). As TR-∆GFP is lacking the US7, US8, and US9 proteins that target components of the type I interferon response pathway [38,39], we performed growth kinetics using the HCMV strain TB40/E. This virus lacks US2-US6, but encodes US7-9. Again, the preincubation of cells with different amounts of DBs showed the same genome replication profiles as infected control cells. These results indicated that DB addition has no effect on HCMV genome replication, irrespective of the DB amount or application timepoint.

To investigate if there was an impact on viral progeny production, the release of viral genomes into the cell culture supernatant was tested by quantitative PCR analysis. As observed for HCMV genome replication, no impact on genome release was found following the concomitant exposure of cells with DBs and KB14 or with DBs and TR-ΔGFP (Figure 1d,e). HFFs that were preincubated with DBs for 2 h prior to infection with TR-ΔGFP (Figure 1e) or TB40/E (Figure 1f) released slightly less progeny than control infected cells, but these differences were not statistically significant. Taken together, these data show that the addition of DBs simultaneously with infection or prior to infection has no effect on HCMV genome replication and only subtle effects on genome release.

### 3.2. Preincubation of HFF with DB Leads to a Reduction in the Release of Infectous Progeny

**Figure 2:**
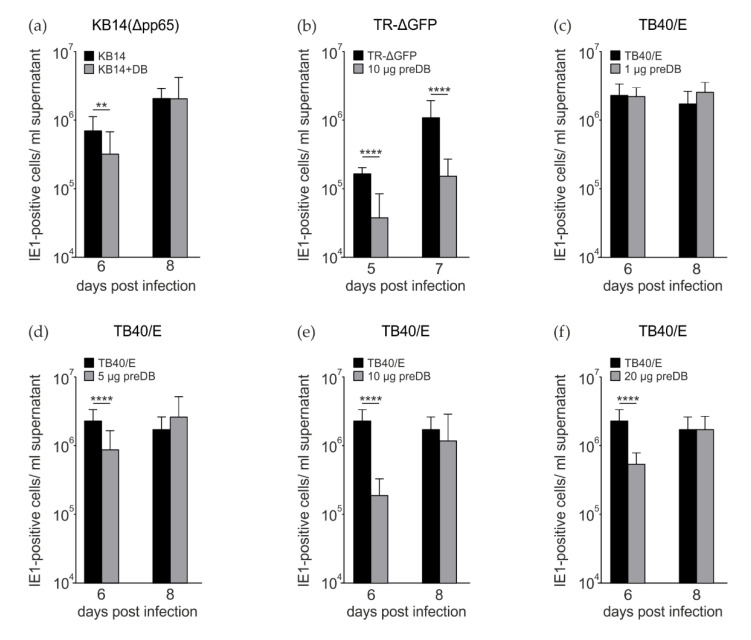
Analysis of HCMV progeny release following coincubation or preincubation of HFFs with DBs. Release of infectious virus from infected HFFs exposed to DBs was determined via IE1 staining following infection of indicator cells in serial dilutions. (**a**) Progeny release from HFFs, infected with 25 genome copies per cell of KB14 (∆pp65) or infected and simultaneously exposed to 10 μg of UV-inactivated DBs of TR-ΔGFP (Δpp65 + DB). (**b**) Progeny release from HFFs, infected with TR-ΔGFP (25 genomes per cell) or preincubated with 10 μg of UV-inactivated DB for 2 h and subsequently infected with TR-ΔGFP (25 genomes per cell, 10 µg preDB). (**c**–**f**) Progeny release from HFFs, infected with TB40/E (moi 0.5) or preincubated with 1 µg, 5 µg, 10 µg, or 20 µg of UV-inactivated DBs for 2 h and then infected with TB40/E (moi 0.5). Supernatants from experiments (**a**–**f**) were collected at the indicated time points. Supernatants were cleared from cell debris by centrifugation and frozen at −80 °C until further use. To determine the levels of infectious virus contained in the cell culture supernatants, serial dilutions of the supernatants were applied to HFFs. Infected cells were visualized using an IE1-specific antibody. Infectivity of viral progeny was determined by counting IE1-positive cells. The data represent mean values ± SD of eight-fold determinations from three independent experiments for each timepoint. ** *p* ≤ 0.01, **** *p* ≤ 0.0001 by Welch’s *t*-test (**a**,**b**) or one-way ANOVA (**c**–**f**).

Measuring viral genome release from HCMV-infected cells serves as a surrogate for virus release. This, however, does not always match the levels of infectious progeny, as it does not control for particle-to-infectivity ratios. Consequently, we also tested for infectivity, using serial dilutions of the culture supernatants and the counting of IE1-positive nuclei on indicator cells as readout [27]. In this instance, coincubation of HFFs with DBs and KB14 led to a statistically significant but moderate reduction in IE1-positive cells, most prominently at 6 dpi (Figure 2a). Next, HFFs were incubated with DB 2 h prior to infection with the pentamer-positive strain TR-ΔGFP (Figure 2b). A significant reduction in progeny release was detectable at both tested time points (Figure 2b). Finally, the experiment was repeated, using the TB40/E stain for infection. In this case, cells were preincubated with different amounts of DBs, prior to TB40/E infection. The experiments confirmed that DB pretreatment leads to a reduction in progeny release at late times after infection. This effect was independent of whether 5 µg, 10 µg or 20 µg DB were applied (Figure 2d–f). No effect was observed when 1 µg of DB was applied (Figure 2c). Taken together, these experiments provided evidence that the pretreatment of HFFs with DBs impaired the downstream release of progeny after HCMV infection to a statistically significant level.

### 3.3. Preincubation of HFF with DB Leads to the Enhancment of Cytopathic Effects and Increased Early Apoptosis

**Figure 3:**
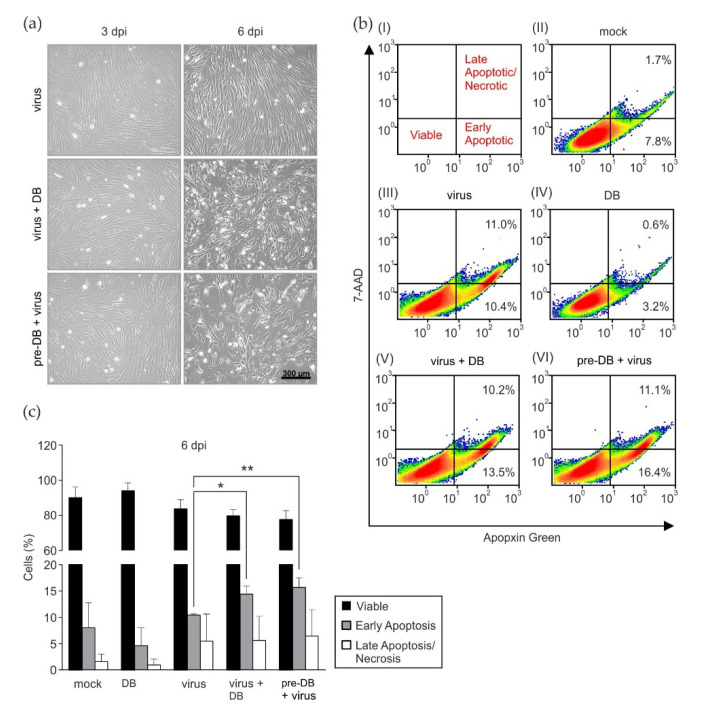
Analysis of apoptotic events following the addition of DBs to infected HFFs. (**a**) HFFs were incubated with 25 genomes/cell of TR-ΔGFP, together with 20 μg of UV-inactivated DBs (virus + DB) of the same strain, or preincubated with 20 μg DB for two hours and subsequently infected (pre-DB + virus). Infected cells served as controls (virus). Following 6 h incubation, the inocula were removed and the cells were washed two times with PBS. Fresh medium was added, and the cells cultivated. The cytopathic effect in these cultures was documented at 3 and 6 d.p.i by light microscopy in 100× magnification. (**b**) Flow cytometry analysis of apoptosis and necrosis in HFFs six days after infection and DB exposure. (**I**) Gating strategy for the different staining patterns used in the FACS analyses. Viable cells appear in the lower left quadrant, early apoptotic cells appear in the lower right quadrant, and late apoptotic/necrotic cells are shown in the upper right quadrant. (**II**) FACS analysis of untreated cells (mock). (**III**) HFFs infected with HCMV strain TR-ΔGFP (50 genomes per cell; virus). (**IV**) Cells incubated with 20 μg UV-inactivated DBs. (**V**) Cells infected and simultaneously treated with 20 μg UV-inactivated DB. (**VI**) Prior to infection HFF were preincubated with 20 μg of UV-inactivated DBs. After 2 h, virus inoculum (HCMV strain TR-ΔGFP; 50 genomes per cell) was added to the cells. The different inocula in (**III**–**VI**) were applied to HFFs for 2 h. Subsequently, fresh medium was added and the cells were incubated at 37 °C and 5% CO_2_ for another 4 h. After a total incubation period of six hours, the inoculum was removed, the cells were washed twice with PBS, and cultivated for six days in fresh medium. For the samples (**III**–**VI**), floating cells from supernatants were combined with adherent cells. Apoptotic and necrotic cells were double labelled with Apopxin Green and 7-AAD. For each condition, 1.5 × 10^5^ cells were recorded. The percentages of cells stained with each dye are shown in the quadrants. (**c**) Quantification of apoptotic/necrotic cells. Data represent mean ±SD of three independent experiments. Comparisons between groups were calculated using an unpaired, two-tailed *t*-test for the indicated group, compared with the appropriate untreated group. * *p* < 0.05; ** *p* < 0.01. 7-AAD, 7-aminoactinomycin D. 7-AAD stains late apoptotic/necrotic cells; Apopxin Green stains apoptotic cells; FACS, Fluorescence-Activated Cell Sorting.

To investigate if preincubation or concomitant incubation of HFFs with DBs had an effect of cell viability following infection, the virally induced cytopathic effect (cpe) was monitored by light microscopy. Upon inspection, it became apparent that both the pretreatment and concomitant treatment of infected cells with DBs led to a marked enhancement of the cpe, particularly at 6 dpi (Figure 3a). The incubation of HFFs with DBs alone showed no alteration of cell growth, compared to the mock infected cells. This suggested that DBs influenced the health of infected cells. To test the hypothesis that DBs are able to induce the apoptosis of infected cells, Fluorescence-Activated Cell Sorting (FACS) analyses for markers of early and late apoptotic cells were performed, as shown in Figure 3b. In three independent experiments, a subtle but statistically significant enhancement of early apoptosis by DBs before and during treatment could be detected (Figure 3c). It should be mentioned that pUL36, which is known to interfere with apoptosis, is conserved in the Towne variant strain, which was used for the experiments. The diminishing effect of DBs alone on apoptosis observed in Figure 3b(IV) was not consistent throughout the three independent experiments. The percentage of early apoptotic cells in DB-treated HFFs was comparable to mock cells in the other two experiments (Figure 3b(II)). The results showed that DBs moderately enhanced the programmed cell death of HCMV-infected cells.

### 3.4. Preincubation of HFFs with DBs Leads to Enhanced Expression of Interferon-Regulated Genes MX1, IFIT3, and ISG15

**Figure 4:**
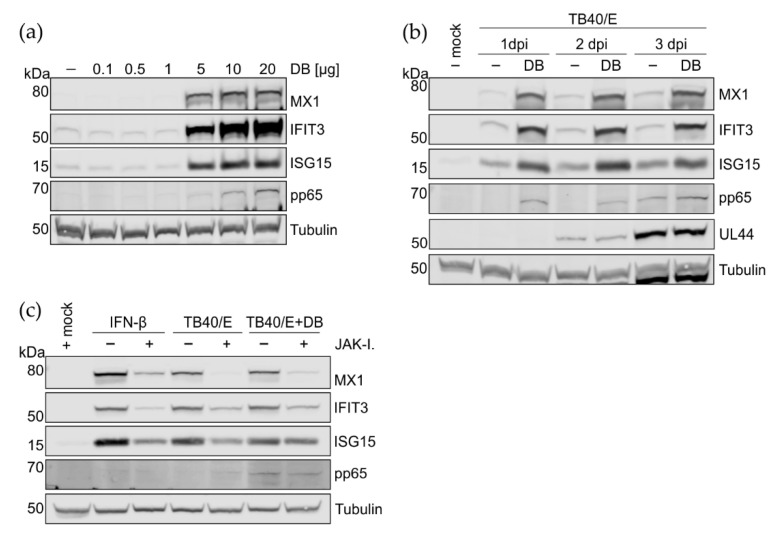
Analysis of the induction of IRGs by DB addition to infected cells and the relevance of the IFN signaling pathway. (**a**) Representative immunoblot analysis of 5 × 10^5^ HFF incubated with increasing amounts of UV-inactivated DBs derived from the HCMV strain TR-∆GFP. Cell lysates were prepared one day after DB application and subjected to SDS-PAGE and immunoblot analyses. Untreated cells (−) served as the control. The membranes were probed with antibodies against MX1, IFIT3 and ISG15. An antibody directed against the viral pp65 protein was used to confirm DB internalization. Tubulin served as the sample loading control. (**b**) HFFs were left uninfected, were infected with TB40/E (moi 0.5) or were pre-treated with 10 µg of UV-irradiated DBs for 2 h before infection with TB40/E (moi 0.5). IRG and viral pp65 and UL44 expression levels were measured up to 3 d.p.i by Western blotting. Tubulin was used as loading control. (**c**) Representative immunoblot analysis of MX1, IFIT3, and ISG15 expression in HFFs upon JAK inhibitor I treatment, compared to the untreated control cells. HFFs were incubated in 5% MEM medium in the presence or absence of JAK inhibitor I (20 μM/mL) for one hour. Afterwards, the cells were infected with the strain TB40/E or infected and simultaneously co-incubated with 10 µg of UV-irradiated DBs. Each sample was further co-treated with JAK inhibitor I for 24 h. Addition of IFN-β (100 U/mL) was used to control the effects of the inhibitor on the JAK-STAT signaling cascade. Pp65 was used as a DB internalization control.

One of the very first defense mechanisms during viral infection is the initiation of the type I interferon (IFN-I) response, mediated by the release of IFN-α and IFN-β. Release of IFN-β by infected fibroblasts leads to the induction of a large number of interferon-regulated genes (IRGs), following IFN-β binding to its cognate receptor on the cell surface. In an initial experiment, we determined the minimal amount of DBs that could trigger IRG expression in the absence of infection. For this, increasing amounts of DB, ranging from 0.1 µg to 20 µg, were applied to HFFs and MX1, IFIT3 and ISG15 expression levels were analyzed by Western blot one day after DB application. Cell lysates were probed using antibodies against IRG MX Dynamin Like GTPase 1 (MX1), Interferon Induced Protein With Tetratricopeptide Repeats 3 (IFIT3) and Interferon Stimulated Gene 15 (ISG15). Amounts of 0.1 µg, 0.5 µg and 1 µg did not elicit any IRG response above the background, whereas 5 µg, 10 µg and 20 µg DB stimulated the expression of IRGs (Figure 4a). The IFN-I response in HCMV infection is met by evasion mechanisms to restrict downstream IRG-expression [40,41,42]. To investigate if the impairment of HCMV infectivity by DB preapplication was related to IFN-I mediated IRG induction, the lysates of infected and DB-pretreated cells were analyzed by immunoblot (Figure 4b). HCMV infection led to a moderate induction of MX1, IFIT3, and ISG15, compared to the mock infected cells, as expected (Figure 4b). Interestingly, DB pretreatment of cells led to a pronounced induction of the three IRGs, compared to HCMV infection. Viral UL44 protein levels used as a control were comparable to those of infected cells (Figure 4b). To demonstrate that this DB-mediated ISG induction is dependent on canonical IFN signaling pathways, we blocked the JAK/STAT signaling cascade by using a JAK inhibitor. In the presence of the inhibitor, IRG expression decreased to similar levels as induced by TB40/E infection (Figure 4c). These data show that the DB pretreatment of cells induces an IFN response that is sustained during subsequent HCMV infection.

## 4. Discussion

DBs were first described many years ago. They have been found in infected culture cells, but also in endothelial cells in vivo [6,19,43]. DBs are highly immunogenic and have thus been denoted as a promising vaccine candidate [13,16,18]. In a previous study, we have shown that DBs when added to cells induce a broad IRG response, which is both IFN-β- and IRF3-dependent [21]. Little attention has been paid, however, thus far to the possible role of these particles during HCMV infection. Compton and colleagues showed that the treatment of peripheral blood mononuclear cells (PBMC) with DBs induces the secretion of inflammatory cytokines interleukin-6 (IL-6) and interleukin-8 (IL-8) [44]. These data, however, did not address the possible impact of DBs on HCMV infection. This is surprising, as laboratory HCMV strains as well as clinically isolated strains differ in the levels of DB synthesis [45]. Considering the fact that DBs might influence the outcome of HCMV infection, both the molecular analysis of infections with one strain as well as comparative experiments between different strains may be biased by the effect of DBs on viral replication.

We have shown that the IFN-β preincubation of HFFs before infection with TR-∆GFP reduced viral genome release by approximately 2 log10 levels [37]. Here, we demonstrate that the preincubation of HFFs with DBs significantly reduces the release of infectious virus from infected cells. These findings thus indicate that DBs sensitize cells against HCMV when they are applied to HFFs before infection. This resembles the findings that the IFN-β exposure of cells during HCMV infection has little effect on viral replication, but pretreatment renders the cells less susceptible to infection.

There are several intrinsic defense mechanisms that protect the cell against viral infection [41]. PML-NBs are nuclear macromolecular protein complexes involved in epigenetic regulation and antiviral defense [46,47,48]. PML-NBs led to the silencing of incoming viral genomes, which is antagonized by the HCMV IE1 protein through the dispersal of these structures. Recent evidence shows that PML-NBs are also involved in the entrapment of incoming viral genomes, as well as of newly assembled capsids [49]. However, we did not detect any impact of DB application on the dispersal of PML-NBs induced by the virus. This does not completely exclude the support of PML-NB function by DBs, as there may be antiviral effects of these structures that are not associated with their dispersal. Still, no impairment of IE1-protein expression was found (Penner et al., unpublished). This argues against an interference of DBs with the gene silencing functions of PML-NBs.

Apoptosis is induced as a defense mechanism of the host cell in response to infection in order to restrict viral spread [50]. HCMV, on the other hand, encodes proteins that block key apoptotic steps to ensure complete viral replication and progeny virus production [42,51,52,53,54,55]. FACS analysis revealed a moderate enhancement of early apoptotic events in HCMV infection following DB treatment. DB application to infected cells, in contrast, induced a marked enhancement of the cytopathic effect upon microscopic inspection. This indicates that mechanisms other than apoptosis are additionally active to mediate the marked cpe, induced by DBs in HCMV-infected cells. Interestingly, HFFs incubated with DB alone did not show any kind of cytopathic alterations, compared to mock-treated cells.

How apoptosis is induced upon DB application to infected cells remains unclear at this point. However, one explanation could imply an effect of IRGs as mediators of apoptosis. Microarray studies have identified IRGs with proapoptotic functions [56]. Some of these IRGs are upregulated in HFFs incubated with DBs [21]. It could be hypothesized that the cumulative effects of these proapoptotic IRGs together with virus infection may cause apoptosis. This may be in accordance with the findings in this study that DB pretreatment leads to an enhancement of IRG expression in infected cells (Figure 4b). In addition, the protein expression levels of these IRGs were sustained up to 3 days in infected cells, whereas they are normally degraded during HCMV infection [42,57,58]. All of this points to a central role of the IFN-I pathway in the restriction of HCMV infection by DBs.

HCMV mutants that do not express the tegument protein pp65 are devoid of DB formation [36]. However, pp65 interferes with DNA sensing, and thus with the downstream induction of IFN-β. To avoid possible bias by using a virus with a defect in IFN/IRG induction, we chose to use a derivative of the HCMV Towne laboratory strain, TR-ΔGFP, which is repaired for pentamer expression or the TB40/E strain for most infection experiments to closely match the wt situation [37]. Both strains express pp65, and thus DB are synthesized in cells infected with this virus. Since infected cells without DB application were always used as a control, the results obtained reflect the impact of additional DB supplemented to the culture. In addition, the effects imposed on infection and the innate response of the cell were predominantly observed when DBs were added to the cultures before the application of the infectious inoculum, and thus reflect the sensitization of cells against HCMV infection.

The role of DBs in vivo remains unclear. We have recently shown that DBs induce multiple IRGs when applied to cells without infection [21]. The results of the work presented here indicate that DBs induce an interferon response in infected cells that has an impact on viral progeny release. This resembles the findings that the pretreatment of cells with IFN-β renders the cells less susceptible to infection [59,60]. It is tempting to assume that infected cells synthesize and release DBs in order to protect cells against infection in a paracrine fashion. This may also apply to more distant sites, as DBs are produced in vivo in endothelial cells [19] and may be transported by the blood stream. However, a legitimate question is whether the amount of DBs released from infected cells is in any way physiological in that it corresponds to the number of virions secreted from the cells. We performed a series of DB purifications via gradient centrifugation without letermovir and also collected the virion bands. From the measurement of the protein content in both fractions, we calculated that approximately about half of the protein mass of DBs is contained in the virion fraction. Because of the different sizes of virions and DB, about four to seven virions are equivalent to one DB. Although we do not know the particle-to-infectivity ratio for HCMV in vivo, even a ten to one ratio would mean that roughly one DB particle would correspond to one to two infectious virions, released from cells. Given that match, the biological relevance of our findings is likely. However, a major limitation of this study is that all experiments were performed in vitro. Thus, the relevance of the findings has to be verified in further studies in animal models.

## 5. Conclusions

In conclusion, DBs have an impact on HCMV progeny production when applied before infection. This is accompanied by the enhanced induction of IRGs and, possibly as a consequence, by the enhanced early apoptosis of infected cells. The underlying molecular mechanisms are unclear at this point. For the analyses of HCMV infection and viral–host interactions, the activities of DBs contained in the inoculum should be considered.

## Data Availability

Not applicable.
